# Wider Than Expected: Geographic Range and Genetic Diversity of a Commercially Farmed and Traded “Cottonii” Seaweed *Kappaphycus malesianus*

**DOI:** 10.1002/ece3.72762

**Published:** 2025-12-23

**Authors:** Ronel T. Aguilar, Bea A. Crisostomo, Jonh Rey L. Gacura, Lourie Ann R. Hinaloc, Michael Y. Roleda

**Affiliations:** ^1^ Algal Ecophysiology Laboratory, The Marine Science Institute, College of Science University of the Philippines Diliman Quezon City Philippines

**Keywords:** conspecific, eucheumatoids, haplotype, *Kappaphycus inermis*, *rbc*L, SSU

## Abstract

This study aims to expand the current knowledge on the distribution and genetic diversity of *Kappaphycus malesianus*, a commercially important carrageenophyte whose cultivation has been limited to the southern Philippines. By broadening the sampling range and using molecular phylogenetic approaches, it seeks to provide a comprehensive overview of the species' genetic structure and diversity in the Philippines. Specimens obtained from four regions across the country were examined for morphological characteristics and subjected to molecular analysis using mitochondrial (*COI*‐5P and *cox*2‐3 spacer), plastid (*rbc*L), and nuclear (SSU) markers. Genetic diversity and haplotype network analyses, along with Bayesian phylogenetic inference, were conducted to assess genetic variation, population structure, and evolutionary relationships among *K. malesianus* populations. These analyses revealed new wild *K. malesianus* populations in Luzon (Sorsogon) and Visayas (Northern Samar, Bohol, and Iloilo), revealing a broader distribution and greater morphological diversity than previously reported. Multiple novel haplotypes were identified, indicating high genetic diversity and a complex population structure shaped by historical gene flow and regional diversification. A key finding is the identification of KMAL‐G, a wild haplotype from Sorsogon and Northern Samar, which genetically links the farmed KMAL‐A to wild Tawi‐Tawi populations, suggesting unrecognized genetic connectivity across these distant regions. Additionally, the close genetic similarity of the unidentified *Kappaphycus* sp. (GUI1) to *K. malesianus* suggests potential conspecificity. Similarly, the high genetic affinity between *K. malesianus* and *K. inermis* suggests a possible species complex. Overall, these findings demonstrate that *K. malesianus* is more geographically widespread and genetically diverse than previously known. The discovery of novel haplotypes highlights the importance of continued genetic surveys for aquaculture improvement. These findings not only provide insights on the domestication history of *K. malesianus* but also advance our understanding of its genetic diversity and phylogeographic distribution in the Philippines.

## Graphical abstract

The study expands the known distribution of *Kappaphycus malesianus* beyond the southern Philippines by documenting new wild populations in Luzon and Visayas through multilocus phylogenetic analyses. High genetic diversity, novel haplotypes, and a key genetic link between farmed and wild populations across distant regions were identified, revealing previously unrecognized regional connectivity. Overall, these findings provide new insights into the domestication history of *Kappaphycus malesianus* and broaden our understanding of its genetic diversity and phylogeographic patterns in the Philippines.

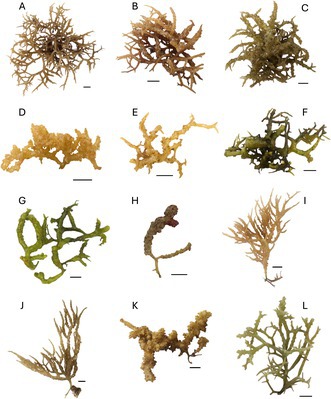

## 1 Introduction

*Kappaphycus malesianus* J. Tan, P.E. Lim & S.M. Phang is a red algal species belonging to the Solieriaceae family. It is one of the cultivated *Kappaphycus* species in the Philippines, following *K. alvarezii* (Doty) L.M.Liao and *K. striatus* (F. Schmitz) L.M.Liao in terms of farming prominence (Crisostomo and Roleda 2024; Dumilag et al. 2023; Tahiluddin et al. 2023). These species are cultivated primarily for their kappa (κ)‐carrageenan, a sulfated polysaccharide that has a wide variety of applications in the food industry, the pharmaceutical sector, and cosmetics. Additionally, its farming serves as a source of income and employment in coastal communities where livelihood opportunities are limited (Ask and Azanza 2002; Bixler and Porse 2011; Hurtado et al. 2013; Valderrama et al. 2013). Among these three species, *K. alvarezii* is the most extensively farmed due to its superior carrageenan yield and quality, followed by *K. striatus* , with *K. malesianus* being the least cultivated (Bui et al. 2019; Tahiluddin et al. 2023). A comparative study by Bui et al. (2019) showed that *K. alvarezii* produces κ‐carrageenan with the highest viscosity, followed by *K. malesianus* and then *K. striatus* . In terms of gelation, *K. malesianus* gels at a slightly higher temperature (23°C) than both *K. alvarezii* and *K. striatus* (19°C), although *K. alvarezii* remains superior in overall carrageenan quality. This finding has important implications for seaweed processors, who have reported inconsistent carrageenan quality, particularly from batches of “cottonii” sourced in the southern Philippines (Roleda, personal communication with seaweed processors). These reports raise the possibility that such lower‐quality batches may be composed of *K. malesianus*, either intentionally or unknowingly farmed.

The cultivation of *K. malesianus* in the Philippines is currently confined to the southern regions of the country (Crisostomo and Roleda 2024; Dumilag et al. 2023; Tahiluddin et al. 2023), with no records of farming in other areas. Furthermore, available information on its broader distribution remains scarce. *K. malesianus* was first documented in Karindingan Island, Malysia, where it was formally identified and classified as a new species (Tan et al. 2014). However, even before its formal taxonomic identification, farmers engaged in seaweed cultivation within the southern regions of the Philippines were already cultivating variants known locally as *Aring‐Aring*, *Halaw*, and *Kuku Belleh*, alongside wild populations known as *Agal batoh*. Later, molecular analyses confirmed that some of these cultivated varieties and wild populations were indeed species of *K. malesianus* (Dumilag et al. 2016, 2023). Although *K. malesianus* is currently reported only from the southern Philippines, its genetic diversity has been shown to be high within this limited range (Dumilag et al. 2023). Nevertheless, this observed diversity does not capture the full extent of its genetic and geographic scope. Given the recent reports of inconsistent carrageenan quality from “cottonii” batches, which may inadvertently include *K. malesianus*, clarifying the actual distribution of the species has become important, not only for taxonomic and ecological purposes but also for improving quality control in seaweed farming and processing. Our recent collections of *Kappaphycus* specimens from different regions in the Philippines suggest that *K. malesianus* may have a broader distribution than previously documented. Building on these findings, this study aims to provide a more comprehensive view of the genetic structure and diversity of *K. malesianus* in the Philippines.

To better understand its distribution and identity, accurate species identification is essential. Generally, *K. malesianus* is identified and distinguished from other species based on morphological characteristics (Tan et al. 2014). However, their morphological features may sometimes overlap with other species, which makes morphology‐based identification of *Kappaphycus* at the species level and its differentiation from other closely related eucheumatoid species difficult (Dumilag et al. 2023). The advent of molecular biology, particularly the development of amplification primers, has been instrumental in the identification of *Kappaphycus* species (Lim et al. 2014; Roleda et al. 2021; Saunders 2005; Saunders and Moore 2013; Tan et al. 2024). In this study, multiple loci that are commonly used for phylogenetic purposes were utilized to assess the genetic structure and diversity of *K. malesianus* in the Philippines. The partial sequences of the 5′ region of the mitochondrial *cytochrome c oxidase I* (*COI*‐5P), *cox*2‐3 intergenic spacer, the chloroplast gene *ribulose‐1,5‐biphosphate carboxylase/oxygenase* large subunit (*rbc*L), and the small subunit ribosomal DNA (SSU), as well as the combined mitochondrial gene sequences (*COI*‐5P + *cox*2‐3) were used to infer the genetic structure and diversity of *K. malesianus* in the country. The morphological characteristics and habitat description of wild *K. malesianus* were also provided.

## 2 Materials and Methods

### 2.1 Sample Collection and Morphological Characterization

A total of 42 wild *Kappaphycus* samples were collected from various regions in the Philippines, including Sorsogon (*n* = 19), Northern Samar (*n* = 16), Bohol (*n* = 6), and Iloilo (*n* = 1). Sample collection was conducted with permission from the Department of Agriculture—Bureau of Fisheries and Aquatic Resources (DA‐BFAR) under Gratuitous Permit no. 0309‐24. Each specimen was photographed with a ruler included for scale. External morphological features were recorded, with emphasis on the color, branching, and texture. For molecular analysis, a small portion (2–3 cm) from the thallus apex was carefully excised, rinsed with sterile distilled water, and preserved in absolute ethanol for subsequent DNA barcoding. A summary of all collected samples is provided in Table A1.

### 2.2 DNA Extraction and Multilocus Identification

Each seaweed specimen was pulverized in a sterile mortar and pestle with the aid of liquid nitrogen. About 20–25 mg of the pulverized tissue was used for genomic DNA isolation following the modified CTAB method adapted by Zuccarello et al. (2006) from Doyle and Doyle (1987). The extracted DNAs were subjected to PCR assays using four barcoding markers, namely *COI*‐5P, *cox*2‐3 spacer, *rbc*L, and SSU, which target the mitochondrial 5′ region of the *cytochrome c oxidase subunit* 1, the *cox*2‐3 intergenic spacer, the *ribulose‐1,5‐bisphosphate carboxylase/oxygenase* large subunit, and the small subunit ribosomal DNA, respectively. The PCR reaction was carried out in a 20 μL reaction volume with 1 μL of 50 ng/μL DNA template, 1× Go Flexi PCR buffer, 2 mM MgCl_2_, 0.2 mM deoxyribonucleotide triphosphates (dNTPs), 0.2 μM of each forward and reverse primer, 1 U GoTaq G2 Flexi DNA Polymerase, and nuclease‐free water. The PCR assay was performed in a 96‐well thermal cycler (TurboCycler Lite, Blue‐Ray Biotech). The amplification of the *COI*‐5P gene using GazF1 and GazR1 (Saunders 2005) was performed with an initial denaturation of 94°C for 4 min; 5 cycles of denaturation at 93°C for 1 min, annealing at 45°C for 1 min, extension at 72°C for 1 min; followed by another 30 cycles of denaturation at 93°C for 30 s, annealing at 50°C for 30 s, extension at 72°C for 30 s, and a final extension at 72°C for 5 min. The same thermal cycling profile was used for the amplification of the *cox*2‐3 intergenic spacer region using cox2‐F and cox3‐R primers (Zuccarello et al. 1999). The amplification of the *rbcL* gene using the F57‐rbcLrevNEW primers (Saunders and Moore 2013) was performed with the following conditions: 95°C initial denaturation for 2 min, followed by 35 cycles of 93°C for 1 min, 47°C annealing for 1 min, 72°C extension for 2 min, and followed by 72°C final extension for 2 min. The amplification of the SSU gene using the G01‐G07 primers (Saunders and Moore 2013) was performed with the following conditions: 94°C initial denaturation for 2 min, followed by 38 cycles of 94°C for 30 s, 50°C annealing for 45 s, 72°C extension for 2 min, and followed by 72°C final extension for 5 min. The amplicons were sent to a sequencing facility in Macrogen Inc., Seoul, South Korea, for standard bidirectional sequencing. The generated raw sequences were subjected to pre‐processing using Geneious Prime (2025) 2025.0.2 (https://www.geneious.com). Forward and reverse sequences were trimmed and aligned using ClustalW (Thompson et al. 2002) at default setting. The resulting alignment was used for species identification through BLASTn analysis (Altschul et al. 1990).

### 2.3 Molecular Analyses

The genetic diversity for each gene marker (*COI*‐5P, *cox*2‐3 spacer, *rbc*L, and SSU) and the combined sequences of two mitochondrial genes (*COI*‐5P + *cox*2‐3 spacer) was computed using DNA Sequence Polymorphism (DnaSP version 6.12.03) (Rozas et al. 2017). The *COI*‐5P sequences of *K. malesianus* from the previous diversity analysis by Dumilag et al. (2023) were included in the final dataset to provide an updated report of diversity based on the *COI*‐5P gene. Haplotype network using TCS version 1.23 (Clement et al. 2000) and phylogenetic analyses using IQ‐TREE (Nguyen et al. 2015) were also conducted for five gene datasets: *COI*‐5P (579 bp), *cox*2‐3 spacer (280 bp), concatenated *COI*‐5P and *cox*2‐3 spacer (859 bp), *rbc*L (1074 bp), and SSU (1518 bp). Additional sequences from NCBI representing the previously known haplotypes for each gene marker were included in the final dataset. Multiple sequence alignment was performed using ClustalW and trimmed to match the length of the shortest sequence. The best‐fit nucleotide substitution models for each dataset were determined using IQ‐TREE based on the Bayesian Information Criterion (BIC) through ModelFinder (Kalyaanamoorthy et al. 2017): HKY + F + I for *COI*‐5P, *cox*2‐3, and for the concatenated mitochondrial dataset, TN + F + I for *rbc*L, and F81 + F for SSU. Maximum likelihood (ML) analyses were performed using the respective best‐fit substitution model for each dataset with 1000 bootstrap replicates, while Bayesian inference (BI) analyses were conducted using MrBayes version 3.2.7a (Ronquist et al. 2012). Markov Chain Monte Carlo (MCMC) simulations were performed using two independent runs, each consisting of four Markov chains, and continued until convergence was reached, as indicated by an average standard deviation of split frequencies below 0.01. A 25% burn‐in was applied to discard initial unstable samples. Phylogenetic trees generated from ML and BI analyses were visualized using FigTree (Rambaut 2022). Bootstrap support values (ML) and posterior probabilities (BI) were mapped onto the final phylogenetic trees to evaluate node confidence.

### 2.4 Haplotype Naming Scheme

Haplotypes were named following Dumilag et al. (2016), using the first letter of the genus and the first three letters of the species (e.g., ***K**appaphycus **mal**esianus* as KMAL). For *COI*‐5P, haplotypes were assigned a letter (e.g., KMAL‐A), while *cox*2‐3 haplotypes were designated by a number (e.g., KMAL‐1). For concatenated mitochondrial gene sequences, both formats were applied (e.g., KMAL‐A1). An exception was made for one specimen from Iloilo, which grouped with a previously identified *COI*‐5P haplotype that did not follow the standard naming scheme. To avoid confusion, this unique specimen, identified as a novel haplotype based on *cox*2‐3, was designated as ILO1. For additional markers such as *rbc*L and SSU, haplotypes were labeled with KMAL, followed by the first two letters of the gene marker (RB for *rbc*L and SS for SSU) and a numerical identifier indicating haplotype number (e.g., KMAL‐RB1, KMAL‐RB2, KMAL‐SS1, KMAL‐SS2).

## 3 Results

### 3.1 Updated Geographic Distribution of *Kappaphycus malesianus* From the Philippines

**FIGURE 1:**
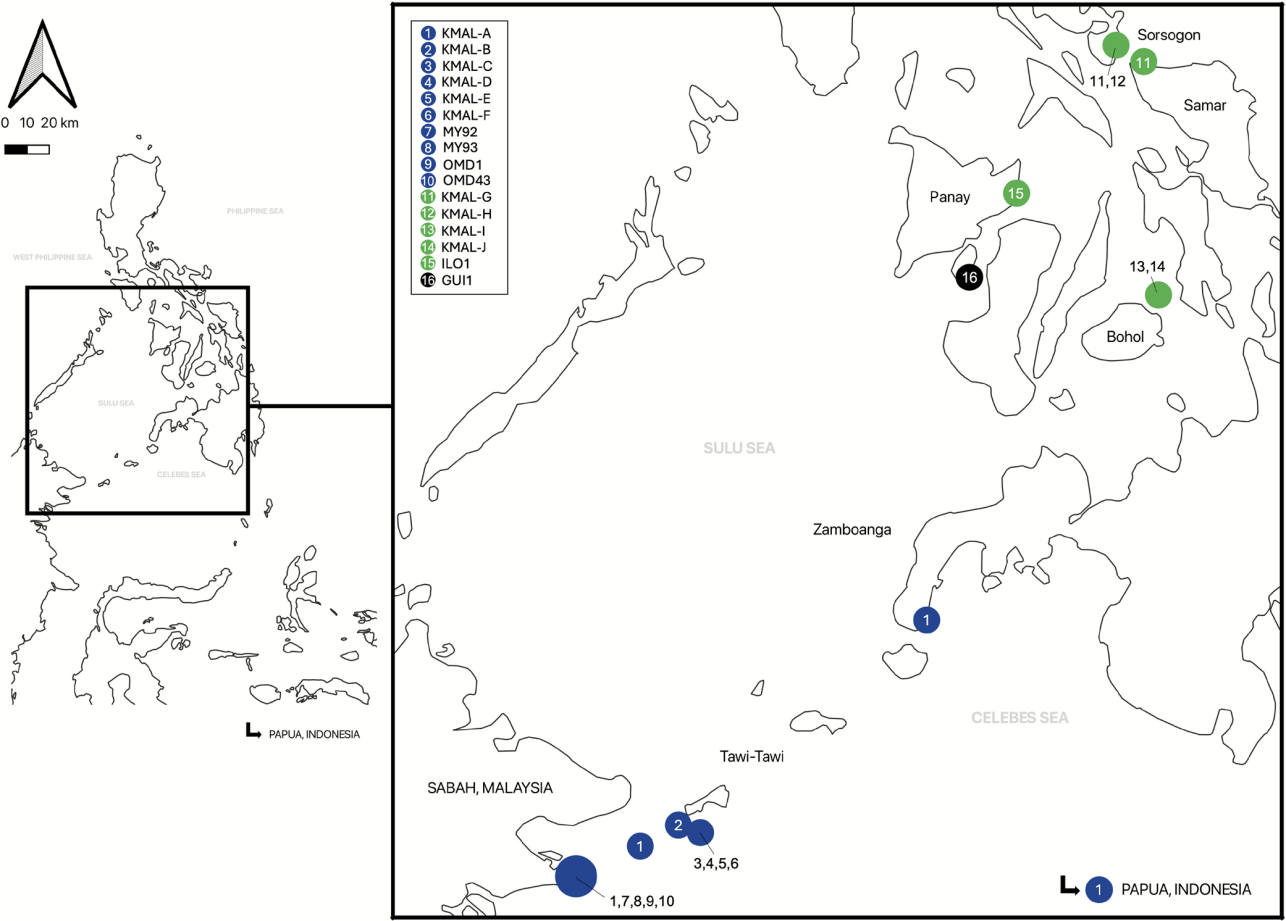
Geographic distribution of *Kappaphycus malesianus* haplotypes across the Philippines, Malaysia, and Indonesia. Green circles represent newly identified haplotypes, blue circles denote previously reported haplotypes, and the black circle indicates the haplotype corresponding to an unidentified *Kappaphycus* species. The numbers within the circles correspond to the haplotype names listed in the legend. The map was created using QGIS version 3.36. Philippine shapefile data were obtained from the Humanitarian Data Exchange (https://data.humdata.org/dataset/cod‐ab‐phl), and site coordinates were sourced from Google Earth.

This study confirmed the presence of *K. malesianus* in regions of the Philippines beyond its previously known southern distribution. A total of 42 *K. malesianus* specimens were identified across four regions: Sorsogon (Southeastern Luzon), Northern Samar (Eastern Visayas), Bohol (Central Visayas), and Iloilo (Western Visayas) (Figure 1). In Sorsogon and Northern Samar, samples were collected from rocky intertidal pools. In Bohol, samples were obtained from a reef flat located 3–4 m deep and approximately 90 km offshore, within the Danajon Bank. In Iloilo, the sample was collected from a sandy to rocky subtidal zone near a remote island in the municipality of Ajuy.

### 3.2 Morphological Characteristics of Wild *Kappaphycus malesianus*

**FIGURE 2:**
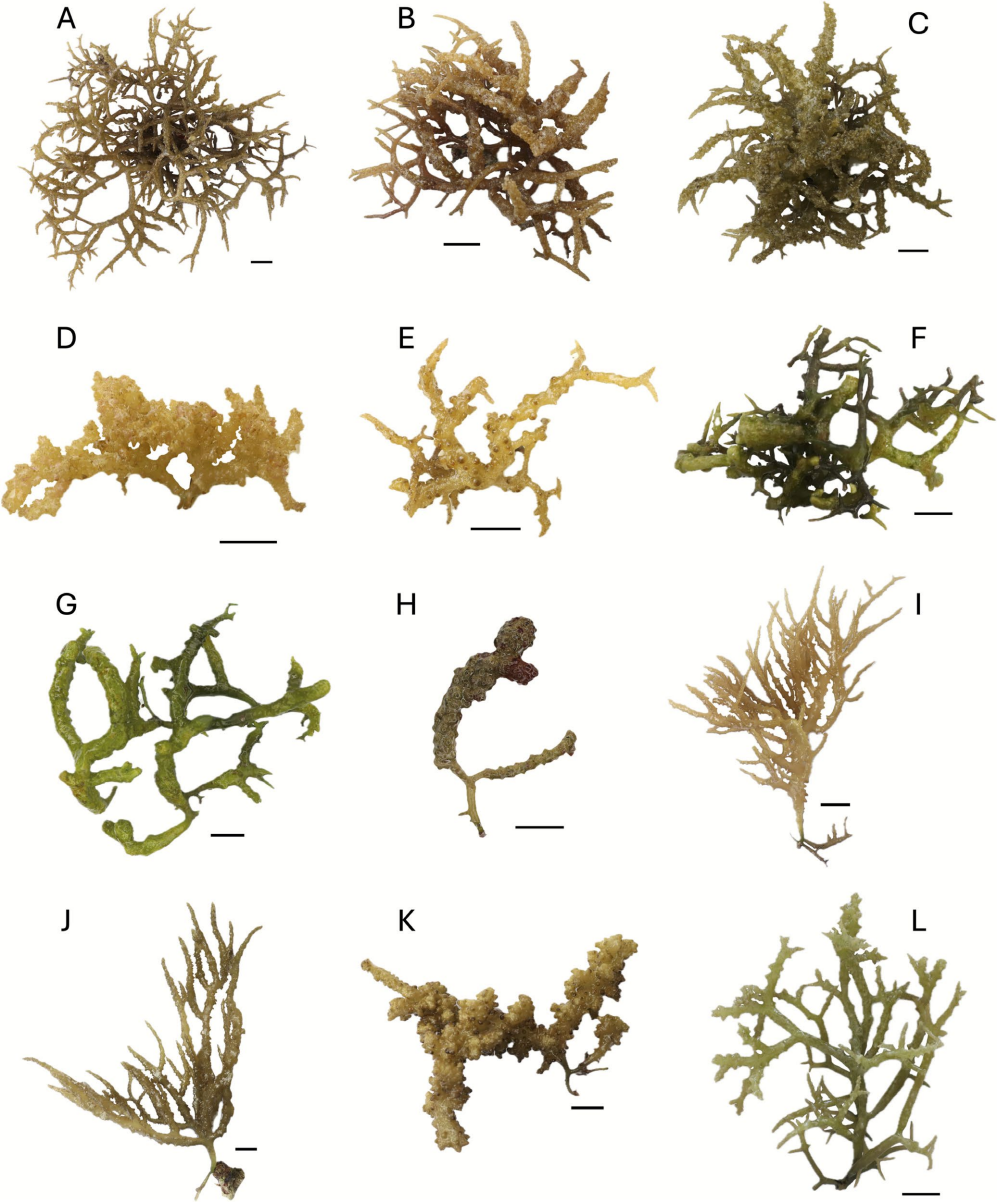
Representative specimens of wild *Kappaphycus malesianus* collected from Sorsogon (A–E), Northern Samar (F–H), Bohol (I–K), and Iloilo (L). Scale bars: 2 cm.

The wild *K. malesianus* specimens examined in this study exhibited diverse color morphotypes, ranging from various shades of brown to green, with distinct branching patterns and thallus textures. Most exhibited a multi‐axis growth form, with mostly sympodial axes, an irregular branching pattern, and a discoid holdfast (Figure 2). Sorsogon specimens (Figure 2A–C) showed the broadest variation, with light to dark brown or greenish brown coloration. They had a decumbent growth habit with bushy, thin branches, characterized by irregular, entangled branching and rough surfaces with nodular protrusions. Some individuals also displayed coarse surface textures (Figure 2D). The cystocarpic specimen (Figure 2E) was golden brown with pronounced surface bumps. Northern Samar samples (Figure 2F–H) had greenish to brown thalli, an irregular open branching pattern, and a more rigid, compact form. Their branches were cylindrical with thicker main axes and twig‐like secondary branches tapering to pointed ends. The cystocarpic specimen (Figure 2H) was darker with reddish pigmentation, enlarged thalli, rounded apices, and a swollen, wrinkled surface due to protrusion of circular cystocarps. Bohol specimens (Figure 2I–K) were brownish to golden in appearance, with erect, pinnate to irregular branches tapering to slender tips. Their thalli were fleshy, cartilaginous, and flexible, with a glossy surface and irregular protrusions or spike‐like formations. The cystocarpic specimen (Figure 2K) was thicker and coarser, with dense cystocarps contributing to its uneven appearance. The Iloilo specimen (Figure 2L) resembled Bohol forms in branching pattern but differed in its greenish coloration. It has cylindrical branches tapering towards the tips, thick primary axes supporting thinner secondary branches, and uneven surfaces with small, pointed protrusions.

### 3.3 Genetic Diversity and Neutrality Tests

The wild *Kappaphycus* specimens obtained from Sorsogon, Northern Samar, Bohol, and Iloilo were conclusively identified as *K. malesianus* based on partial sequences of four genetic markers: *COI*‐5P, *cox*2‐3, *rbc*L, and SSU. Generally, the genetic diversity of *K. malesianus* populations from the Philippines exhibited varying levels of variation across different loci, with mitochondrial genes (*COI*‐5P and *cox*2‐3) displaying the highest diversity and chloroplast (*rbc*L) and nuclear (SSU) genes being highly conserved (Table 1). The highest number of informative sites was observed in the combined dataset (*COI*‐5P + *cox*2‐3) (*S* = 13), making it the most variable. On the other hand, *rbc*L (*S* = 4) and SSU (*S* = 2) have very few segregating sites, indicating low mutation rates. The highest nucleotide diversity (*π*) was observed in the *cox*2‐3 (*π* = 7.65 × 10^−3^), while SSU (*π* = 0.36 × 10^−3^) has the lowest. Highest average nucleotide differences (*k*) were observed in the combined mitochondrial genes (*k* = 4.08130), while lowest in SSU (*k* = 0.54444). The *COI*‐5P has the highest number of haplotypes (*h* = 10), followed by *COI*‐5P + *cox*2‐3 (*h* = 9), reinforcing the high genetic diversity in these mitochondrial regions. In contrast, *rbc*L and SSU showed lower diversity, with only two and three haplotypes, respectively, indicating that these loci are more conserved. Highest haplotype diversity was also observed in *COI*‐5P (hd = 0.710), followed by *cox*2‐3 (hd = 0.651) and then the combined mitochondrial genes (hd = 0.642), while *rbc*L (hd = 0.006) has the lowest, indicating very little variation. Meanwhile, neutrality tests revealed negative *F*s and *D* values using mitochondrial genes, while positive values using the *rbc*L and SSU genes, but their *P* values were not significant.

**TABLE 1:** Summary of genetic diversity indices and neutrality test results for each DNA marker used for the identification of *Kappaphycus malesianus* from the Philippines.

Locus	Aligned length (bp)	*n*	Segregating sites (S)		*π* (SD)	*k*	*h*	hd (SD)	Fu's *Fs*		Tajima's *D*	
			SS	PIS					*F*s	*p* value^a^	*D*	Significance
*COI*‐5P	579	81	4	8	0.00340 (0.00047)	1.97099	10	0.710 (0.032)	−0.23192	0.35035	−0.50481	*p* > 0.10; ns
*cox*2‐3	280	47	3	7	0.00765 (0.00081)	2.14246	7	0.651 (0.057)	0.01914	0.64300	−0.15542	*p* > 0.10; ns
*COI*‐5P + *cox*2‐3	859	42	5	13	0.00475 (0.00069)	4.08130	9	0.642 (0.073)	−0.10882	0.74600	−0.07889	*p* > 0.10; ns
*rbc*L	1074	41	0	4	0.00108 (0.00029)	1.16098	2	0.006 (0.078)	0.06747	0.96345	0.57396	*p* > 0.10; ns
SSU	1518	36	0	2	0.00036 (0.00006)	0.54444	3	0.510 (0.064)	0.08680	0.59475	0.25036	*p* > 0.10; ns

Abbreviations: *π*, nucleotide diversity; *h*, number of haplotypes; hd, haplotype diversity; *k*, average number of nucleotide differences; *n*, number of sequences; ns, not significant; PIS, Parsimony informative sites; SD, standard deviation; SS, singleton variable sites.

^a^ Should be regarded as not significant if *p* > 0.05.

### 3.4 Haplotype Network and Phylogenetic Analyses

**FIGURE 3:**
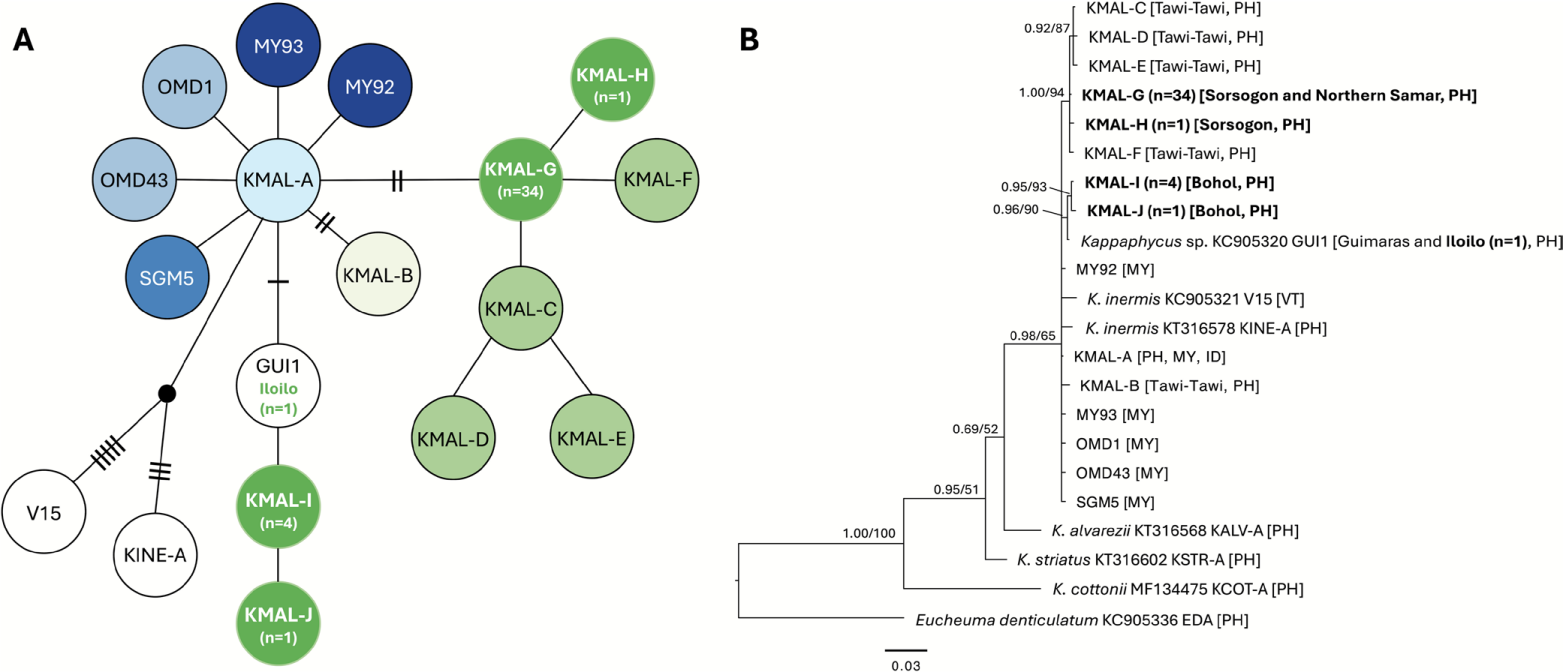
TCS haplotype network (A) and Bayesian inference phylogenetic tree (B) of *Kappaphycus malesianus* from the Philippines and neighboring countries based on *COI*‐5P gene sequences. The TCS haplotype network presents unique haplotypes as circles, with hatch marks indicating the number of nucleotide differences between them. Color patterns for previously identified haplotypes follow the TCS figure of Dumilag et al. (2023), while newly discovered haplotypes are highlighted in bright green. The *n* denotes the number of sequences generated in this study. The BI phylogenetic tree shows posterior probability/bootstrap support values at major nodes, with sequences generated in this study shown in bold. ID, Indonesia; MY, Malaysia; PH, Philippines; VT, Vietnam.

Haplotype network analysis of *K. malesianus* populations based on *COI*‐5P gene sequences (*n* = 55; 41 sequences from this study and 14 sequences of previously identified haplotypes including GUI1 for *Kappaphycus* sp., and KINE‐A and V15 for *K. inermis* ) revealed 15 haplotypes. Nine of which are restricted to the Philippines, five to Malaysia, and one haplotype, KMAL‐A, present in both countries as well as in Indonesia, representing the haplotype of commercially farmed *K. malesianus*. Previously, there were 11 confirmed *COI*‐5P *K. malesianus* haplotypes, six (KMAL‐A to F) of which were reported in the Philippines. This study has detected four additional haplotypes designated as KMAL‐G, KMAL‐H, KMAL‐I and KMAL‐J (Figure 3A). Haplotype KMAL‐G represents wild *K. malesianus* populations from Sorsogon and Northern Samar, while KMAL‐H represents a unique haplotype in Sorsogon. Haplotypes KMAL‐I and KMAL‐J represent wild *K. malesianus* from Bohol differing by only a single nucleotide. In addition, another sample obtained from Iloilo shared a haplotype group with GUI1, an unidentified *Kappaphycus* sp. collected from Guimaras, Philippines. Notably, KINE‐A and V15 are separated by several mutational steps from the KMAL‐A haplotype. Overall, the network exhibited a star‐like topology, with KMAL‐A positioned as the central haplotype and other haplotypes from Malaysia and Philippines radiating outward. As a note on sequence alignment: to standardize the alignment across all *COI*‐5P sequences, the final gene length was trimmed to 579 bp to match the shortest sequence in the dataset. This adjustment caused the previously identified haplotypes KMAL‐B (Tawi‐Tawi, Philippines) and MY92 (Malaysia) to become identical to KMAL‐A, merging them into a single haplotype. To preserve the clarity of haplotype diversity and highlight the discovery of novel variants, the original haplotype network structure based on Dumilag et al. (2023) was retained, with modifications emphasizing the new haplotypes identified in this study. For the phylogenetic analysis, however, the original 602 bp length of previously published sequences was retained, while the newly generated sequences were analyzed at 579 bp. The list of sequence accession numbers and data sources is provided in Table A1. Based on the phylogenetic analysis inferred from the *COI*‐5P gene (Figure 3B), the earliest diverging clade includes KMAL‐A, KMAL‐B, and several wild Malaysian haplotypes. Additionally, a distinct and well‐supported subclade is formed by KMAL‐C, KMAL‐D, and KMAL‐E, all collected from Tawi‐Tawi, suggesting localized diversification in this region. Meanwhile, KMAL‐G, which appears to be the most abundant haplotype in Sorsogon and Northern Samar, is nested within a broader clade that includes KMAL‐F, KMAL‐H, and other haplotypes from Tawi‐Tawi, suggesting that these lineages likely share a common ancestor despite being from geographically separate regions. Similarly, KMAL‐I and KMAL‐J, both from Bohol, cluster together with GUI1 from Guimaras, while the specimen from Iloilo also groups with GUI1, implying a possible shared genetic origin or connectivity between these regions. Furthermore, haplotypes KINE‐A (Ilocos Norte, Philippines) and V15 (Vietnam), both identified as *K. inermis* , clustered within the *K. malesianus* clade. Their relatively long branch lengths and position suggest that they may represent ancestral or geographically isolated lineages.

**FIGURE 4:**
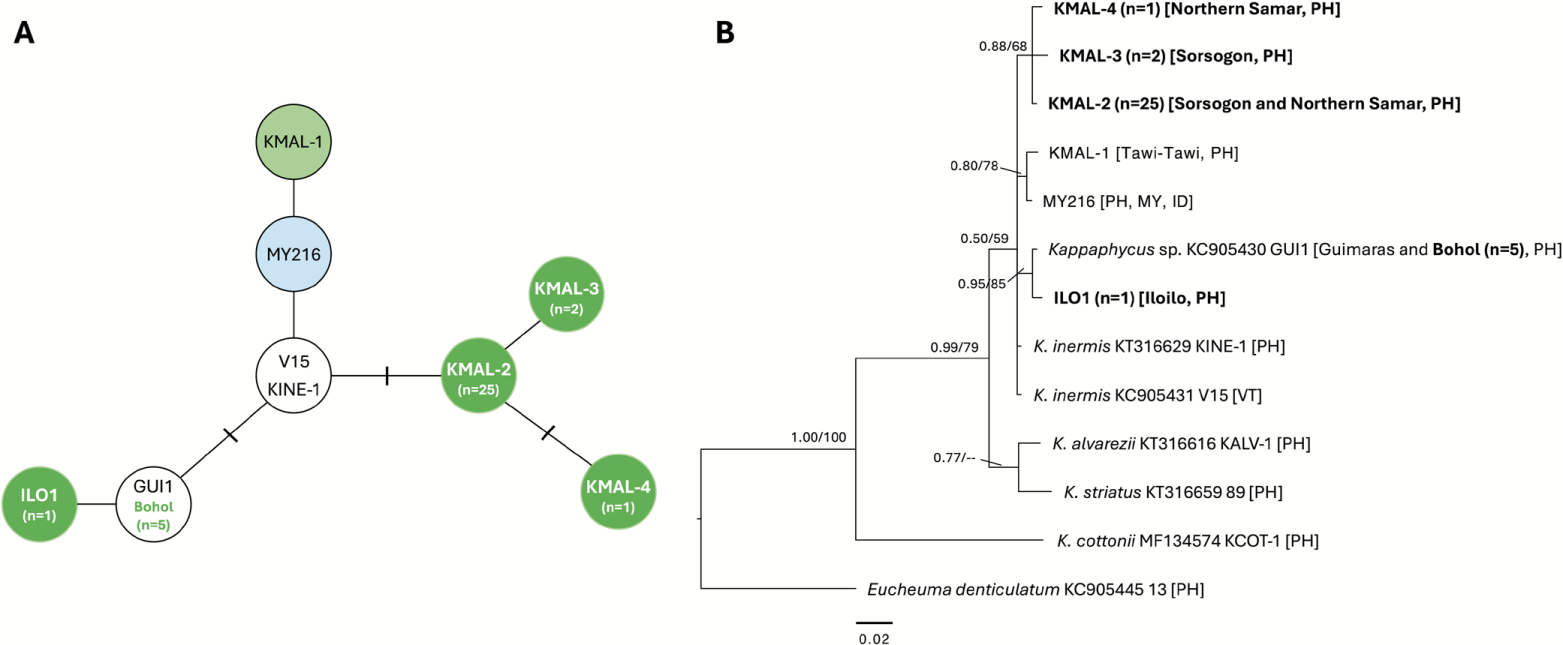
TCS haplotype network (A) and Bayesian inference phylogenetic tree (B) of *Kappaphycus malesianus* from the Philippines and neighboring countries based on *cox*2‐3 intergenic sequences. The TCS haplotype network presents unique haplotypes as circles, with hatch marks indicating the number of nucleotide differences between them. Newly discovered haplotypes are highlighted in bright green circles. The *n* denotes the number of sequences generated in this study. The BI phylogenetic tree shows posterior probability/bootstrap support values at major nodes, with sequences generated in this study shown in bold. ID, Indonesia; PH, Philippines; MY, Malaysia; VT, Vietnam.

Haplotype network analysis based on *cox*2‐3 spacer sequences (*n* = 39; 34 sequences of wild *K. malesianus* from this study and an additional five sequences of previously identified haplotypes including GUI1, KINE‐1, and V15) revealed 6 haplotypes (Figure 4A). These consisted of four newly identified haplotypes designated as KMAL‐2, KMAL‐3, KMAL‐4, and ILO1, alongside two previously reported haplotypes, MY216 and KMAL‐1. Haplotype MY216 corresponds to the KMAL‐A haplotype based on the *COI*‐5P gene, which represents the haplotype of commercially farmed *K. malesianus* populations. Haplotype KMAL‐2 is predominant in Sorsogon and Northern Samar, while KMAL‐3 and KMAL‐4 were identified as unique haplotypes from Sorsogon and Northern Samar, respectively. ILO1 represents a unique haplotype found exclusively in Iloilo, while populations from Bohol clustered together with the previously identified GUI1 haplotype from Guimaras. Notably, KINE‐1 and V15 grouped as a single haplotype, exhibiting 100% homology based on the *cox*2‐3 gene region. Meanwhile, phylogenetic analysis of *K. malesianus* populations (Figure 4B) showed that MY216, a haplotype found in the Philippines, Malaysia, and Indonesia, formed a distinct clade along with KMAL‐1 from Tawi‐Tawi. KMAL‐2, the predominant haplotype in Sorsogon and Northern Samar, formed a well‐supported clade with KMAL‐3 and KMAL‐4, suggesting diversification within these two regions. Similar to *COI*‐5P, wild *K. malesianus* populations from Iloilo (ILO1), Bohol, and Guimaras (GUI1) clustered together, with specimens from Bohol assigned to the GUI1 haplotype. This clustering suggests potential genetic connectivity or an ongoing gene flow among these populations. Moreover, the *K. inermis* haplotypes (KINE‐1 and V15) represent the earliest diverging lineages within the *K. malesianus* clade. Consistent with the *COI*‐5P phylogeny, their close phylogenetic affinity to *K. malesianus* indicates a high degree of genetic similarity between the two species.

**FIGURE 5:**
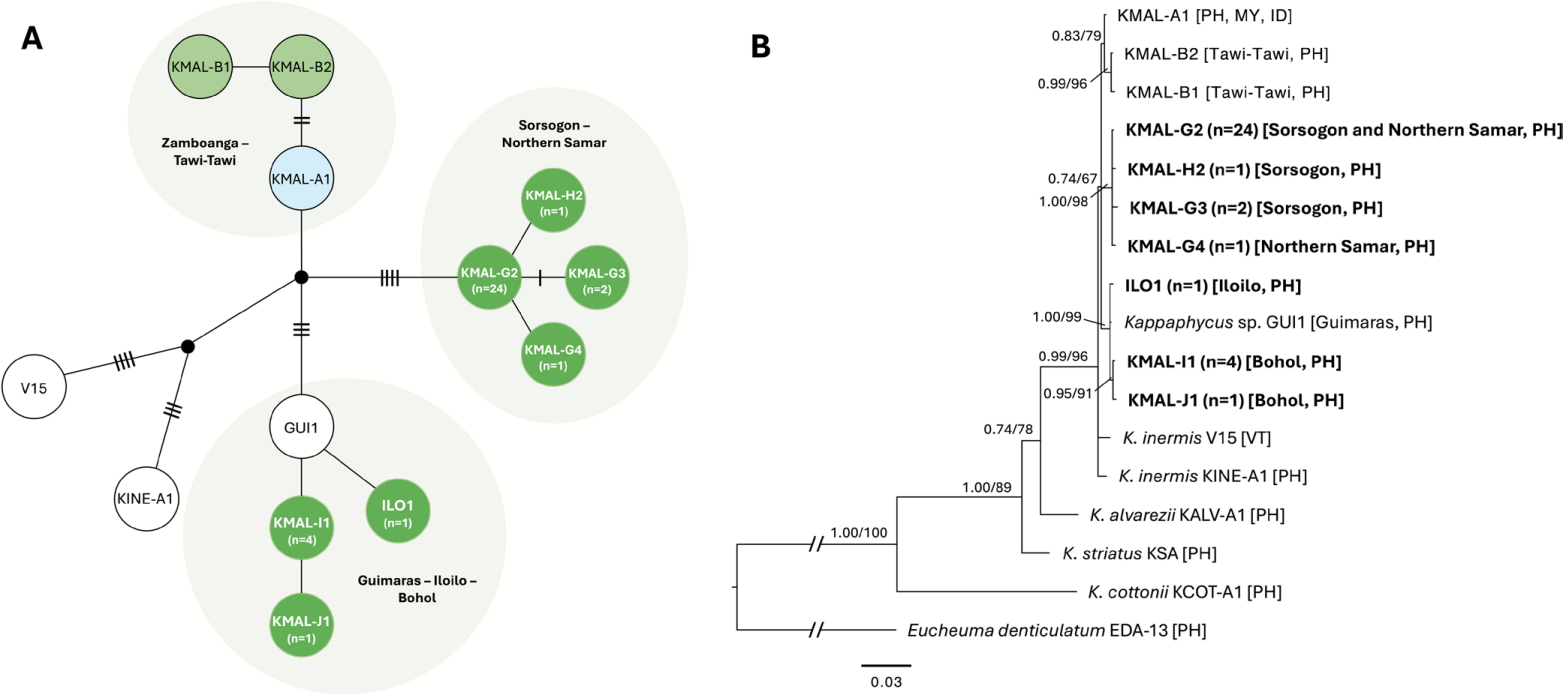
TCS haplotype network (A) and Bayesian inference phylogenetic tree (B) of *Kappaphycus malesianus* from the Philippines and neighboring regions based on concatenated *COI*‐5P and *cox*2‐3 spacer gene sequences. The TCS haplotype network presents unique haplotypes as circles, with hatch marks indicating the number of nucleotide differences between them. Newly discovered haplotypes are highlighted in bright green circles. The *n* denotes the number of sequences generated in this study. The BI phylogenetic tree shows posterior probability/bootstrap support values at major nodes, with sequences generated in this study shown in bold. IND, Indonesia; MY, Malaysia; PH, Philippines; VT, Vietnam. Note on sequence data availability: Haplotypes KMAL‐C, D, E, and F (previously identified based on *COI*‐5P) were excluded from the concatenated data analysis due to the unavailability of their corresponding *cox*2‐3 sequences in public databases. Malaysian haplotypes were also excluded for the same reason, as *cox*2‐3 sequence data were not available.

The haplotype network analysis based on the combined mitochondrial gene sequences (*COI*‐5P + *cox*2‐3 spacer) identified 13 haplotypes, including seven newly discovered haplotypes: KMAL‐G2, KMAL‐G3, KMAL‐G4, KMAL‐H2, KMAL‐I1, KMAL‐J1, and ILO1. Previously identified haplotypes include KMAL‐A1, KMAL‐B1, KMAL‐B2, GUI1, KINE‐A1, and V15. The network revealed three major haplogroups corresponding to distinct geographic regions: (1) the Zamboanga–Tawi‐Tawi (southern region) group (KMAL‐A1, KMAL‐B1, and KMAL‐B2), (2) the Sorsogon–Northern Samar group (KMAL‐G2, KMAL‐G3, KMAL‐G4, and KMAL‐H2), and (3) the Guimaras–Iloilo–Bohol group (GUI1, ILO1, KMAL‐I1, and KMAL‐J1) (Figure 5A). Among these, KMAL‐A1, originally identified from Zamboanga del Sur, Philippines, represents the haplotype of cultivated *K. malesianus*. This haplotype is also present in Malaysia and Indonesia, indicating that it is not geographically restricted to the Philippines. KMAL‐B1 and KMAL‐B2 from Tawi‐Tawi are closely related to KMAL‐A1, differing by only two to three mutational steps. The Sorsogon–Northern Samar group represents the most abundant and genetically cohesive group in the network. KMAL‐G2 acts as the central haplotype in this group, with the other haplotypes radiating outward by one to two mutational steps. The haplotypes from Guimaras, Iloilo, and Bohol formed a separate cluster connected through the GUI1 haplotype, with only one to two nucleotide differences separating them. The phylogenetic tree supports the genetic structure observed in the haplotype network (Figure 5B). The farmed haplotype, KMAL‐A1, clustered with KMAL‐B1 and KMAL‐B2 from Tawi‐Tawi, forming the earliest diverging clade within *K. malesianus*. In contrast, haplotypes KMAL‐G2, KMAL‐G3, KMAL‐G4, and KMAL‐H2 clustered into a separate, well‐supported clade, representing the most recently diverged lineage. This pattern suggests that *K. malesianus* populations in Sorsogon and Northern Samar have undergone recent regional diversification. The close phylogenetic placement of GUI1 with KMAL‐I1, KMAL‐J1, and ILO1 also indicates a strong genetic relationship among populations from Guimaras, Bohol, and Iloilo. Notably, the *K. inermis* haplotypes (KINE‐A1 and V15) are positioned outside the main *K. malesianus* clade. However, their relatively short distance from the base of the *K. malesianus* group suggests a close evolutionary relationship between the two species.

**FIGURE A1:**
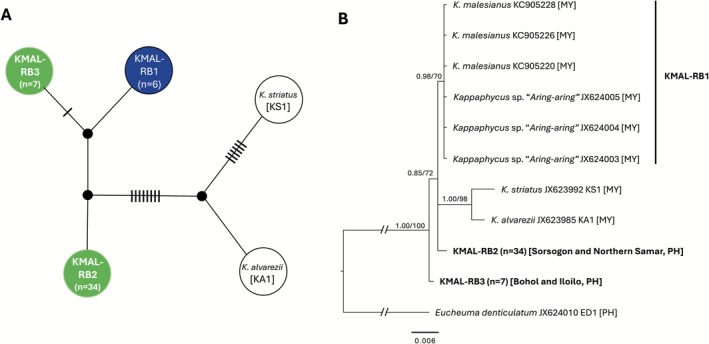
TCS haplotype network (A) and Bayesian inference phylogenetic tree (B) of *Kappaphycus malesianus* from the Philippines and Malaysia based on *rbc*L gene sequences. The TCS haplotype network presents unique haplotypes as circles, with hatch marks indicating the number of nucleotide differences between them. Newly discovered haplotypes are highlighted in bright green circles. The “n” denotes the number of sequences generated in this study. The BI phylogenetic tree shows posterior probability/bootstrap support values at major nodes, with sequences generated in this study shown in bold. MY, Malaysia; PH, Philippines.

Haplotype network analysis based on *rbc*L gene sequences revealed three distinct haplotypes designated as KMAL‐RB1, KMAL‐RB2, and KMAL‐RB3, representing *K. malesianus* populations from different geographic regions (Figure A1A). Haplotype KMAL‐RB1 represents populations from Malaysia, consisting of *K*. *malesianus* sequences that had not been previously assigned a haplotype name. In contrast, haplotypes KMAL‐RB2 and KMAL‐RB3 represent Philippine populations, specifically from Sorsogon and Northern Samar (RB2) and Bohol and Iloilo (RB3), respectively. These two haplotypes are separated from KMAL‐RB1 by a few mutational steps. This network pattern is consistent with the phylogenetic analysis which shows the genetic distinction of the three haplotypes (Figure A1B).

**FIGURE A2:**
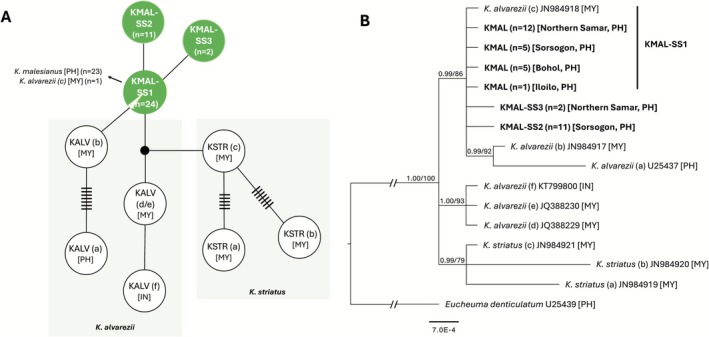
TCS haplotype network (A) and Bayesian inference phylogenetic tree (B) of *Kappaphycus malesianus* and *Kappaphycus alvarezii* based on SSU gene sequences. The TCS haplotype network presents unique haplotypes as circles, with hatch marks indicating the number of nucleotide differences between them. Newly discovered haplotypes are highlighted in bright green circles. The “n” denotes the number of sequences generated in this study. The BI phylogenetic tree shows posterior probability/bootstrap support values at major nodes, with sequences generated in this study shown in bold. Assigned letters in parentheses are representative labels and should not be interpreted as haplotype designations. KALV, *Kappaphycus alvarezii*; KMAL, *Kappaphycus malesianus*; KSTR, *Kappaphycus striatus*; IN, India; MY: Malaysia; PH: Philippines.

In addition to the *rbc*L analysis, haplotype network based on SSU gene sequences revealed three haplotypes, designated as KMAL‐SS1, KMAL‐SS2, and KMAL‐SS3 (Figure A2A). KMAL‐SS1 consists of populations from Sorsogon, Northern Samar, Bohol, and Iloilo, along with one previously identified *K. alvarezii* sequence (JN984918) from Malaysia reported by Thien et al. (2011f). In contrast, KMAL‐SS2 is exclusive to Sorsogon, while KMAL‐SS3 is limited to Northern Samar. To date, there are no publicly available SSU sequences for *K. malesianus* in the NCBI database. The SSU sequences presented in this study are the first records for *K. malesianus* from the Philippines. Although BLAST searches consistently yield *K. alvarezii* as the closest match, with high sequence similarity, we eliminate the possibility of misidentification because the three other independent genetic markers consistently assigned them to *K. malesianus*. One possible explanation is the slow evolutionary rate of the SSU gene, which limits its ability to resolve closely related species. Similar to ITS, the SSU is unsuitable to discriminate *K. alvarezii* and *K. malesianus* (Tan et al. 2024). In the present analysis, the inclusion of available *K. alvarezii* SSU sequences revealed that KMAL haplotypes clustered closely with KALV haplotypes, separated by only a few mutational steps (Figure A2A). This further suggests that the SSU marker may not provide sufficient resolution for clear species‐level or intraspecific differentiation, particularly between the *K. malesianus* and *K. alvarezii* species. Meanwhile, the phylogenetic analysis revealed a well‐supported separation between *K. malesianus* from the Philippines and the other species such as *K. alvarezii* and *K. striatus* (Figure A2B). *Kappaphycus malesianus* sequences from Northern Samar, Sorsogon, Bohol, and Iloilo formed a clade that also included the Malaysian *K. alvarezii* (JN984918). Within this clade, haplotypes KMAL‐SS2 (Sorsogon) and KMAL‐SS3 (Northern Samar) appeared as distinct lineages. While the SSU marker can support broad genetic classification, it may be insufficient on its own for distinguishing recently diverged taxa or detecting intraspecific variation.

## 4 Discussion

### 4.1 Morphological Diversity

This study revealed that *K. malesianus* has a wider geographic range and greater genetic diversity than previously recognized. The morphological diversity of *K. malesianus* also appears to be more extensive than previously documented. While most of the observed traits align with earlier descriptions (Dumilag et al. 2023; Tan et al. 2014), several specimens in this study exhibited unique branching patterns, characterized by a compact and entangled appearance, along with a distinct uneven and textured surface present in both non‐fertile and cystocarpic plants. Notably, a cystocarpic specimen from Northern Samar closely resembled the previously described morphology of a female *K. inermis* , particularly having inflated, wrinkled apical branches caused by the protrusion of dark, circular cystocarps (Dumilag and Lluisma 2014). These observations highlight the broad morphological range of *K. malesianus* and morphological overlap with closely related species. Furthermore, notable regional variations in morphology were observed, suggesting that environmental factors may have influenced these differences, a pattern consistent with the known morphological plasticity of *Kappaphycus* species (Doty 1988; Leliaert et al. 2014). Interestingly, despite these regional morphological differences, some populations from Sorsogon and Northern Samar shared identical haplotypes, and those from Bohol and Iloilo exhibited haplotypes with high genetic homology. This suggests that morphological diversity in *K. malesianus* likely results from complex interactions between genetic and environmental factors, where genetically similar or even identical individuals may display different phenotypes in response to local environmental conditions.

### 4.2 Genetic Diversity and Phylogeographic Distribution of *Kappaphycus malesianus*

The results of genetic analysis based on the mitochondrial *COI*‐5P gene are consistent with the earlier work of Dumilag et al. (2023), while also revealing additional haplotypes with greater haplotype diversity. In contrast, the low genetic diversity observed in *rbc*L and SSU indicates strong conservation in these genes, which makes them useful for species identification but not for detecting gene variability within populations. While mitochondrial loci showed slightly negative Fu's *F*s and Tajima's *D* values, which are often associated with population expansion, these were not statistically significant. However, this requires further verification by increasing the sample size to enhance statistical power and verify these findings. Nevertheless, the haplotype network based on *COI*‐5P presents a more complex picture. The overall network exhibits a star‐like topology centered around KMAL‐A, especially because multiple Malaysian haplotypes are closely connected to it. This radiating pattern is a typical signature of recent population expansion. However, when considering the Philippine populations alone, the network topology suggests regional structuring, setting apart the Sorsogon—Northern Samar—Tawi‐Tawi group from the Guimaras—Iloilo—Bohol cluster. The phylogenetic clustering of wild *K. malesianus* haplotypes from Sorsogon and Northern Samar with those from Tawi‐Tawi, despite their geographical distance, suggests a possible historical genetic connectivity between these regions. In the previous study of Dumilag et al. (2023), there was a missing haplotype between the farmed KMAL‐A and the wild haplotypes from Tawi‐Tawi (KMAL‐C to KMAL‐F). This present study reveals that this gap is bridged by KMAL‐G, a wild haplotype predominant in Sorsogon and Northern Samar, which serves as a genetic link between the farmed haplotype KMAL‐A and wild populations from Tawi‐Tawi. Based on its phylogeographic position, it is possible that KMAL‐G originated from the southern region and was later translocated northward during the early phase of *Kappaphycus* farming. Supporting this interpretation is the documented transfer of an unidentified *Kappaphycus* species referred to as “*Aring‐Aring*” from Pasiagan, Bongao, Tawi‐Tawi to the demonstration farms operated by the National Seaweed Training and Development Center (NSTDC) in Dancalan, Bulusan, Sorsogon in March 2002 (Villanueva et al. 2011). This deliberate translocation may have facilitated the movement of genetic materials across these two distant regions. It is also worth noting that most of the Sorsogon samples collected in this study were collected just a few meters from the NSTDC demonstration farms. Another possibility is that the common ancestor of southern *K. malesianus* populations and those from Sorsogon—Northern Samar once had a wider historical distribution, and what we are seeing today is a remnant of a once connected population that has since become regionally isolated. It is also possible that intermediate populations may have existed or still exist between these regions but remain unsampled. To resolve this pattern, it is recommended to have expanded sampling across Visayas and Mindanao regions as it may reveal additional haplotypes that could clarify the genetic connections across these regions. Nevertheless, the discovery of this connecting haplotype (KMAL‐G) in regions far north of Tawi‐Tawi challenges the previous assumption that *K. malesianus* is restricted to the southern Philippines.

The concatenated mitochondrial loci (*COI*‐5P + *cox* 2–3) further improved the resolution of the genetic structure of *K. malesianus*, delineating regional haplogroups and highlighting both recent expansion patterns within haplogroups. The haplotype network exhibited a branching topology, indicative of regional diversification and the presence of long‐standing, geographically structured wild *K. malesianus* populations across the Philippines. Based on this structure, three major regional haplogroups were identified: (1) the Zamboanga—Tawi‐Tawi (southern region) group, (2) the Sorsogon—Northern Samar group, and (3) the Guimaras—Iloilo—Bohol group. In the Sorsogon—Northern Samar group, the presence of shared haplotypes may be explained by the influence of local oceanographic currents flowing through the San Bernardino Strait—a passage that separates the Bicol Peninsula (where Sorsogon is located) from Samar Island (where Northern Samar is located) (Gordon et al. 2011; Jones et al. 2011). This strait allows the water exchange between the Philippine Sea and surrounding coastal areas, potentially facilitating the natural dispersal of vegetative fragments or spores of *K. malesianus*, which may have played a role in the genetic connectivity observed between these regions. Similarly, the genetic similarity observed within the Guimaras—Iloilo—Bohol group may also be attributed to regional current systems. One possible pathway involves the Mindanao Current, which brings water into the Bohol Sea through the Surigao Strait. This water mass, including inflows from the western Pacific, continues westward through the Dipolog Strait into the Sulu Sea, influencing adjacent straits, including the Guimaras and Iloilo Straits (Gordon et al. 2011). These straits, situated within the Sulu Sea circulation system, are likely shaped by this broader oceanographic flow, which may have enabled the dispersal of *K. malesianus* propagules and contributed to the genetic connectivity observed among these regions. In contrast, the Southern region group contains more basal haplotypes and represents the earliest diverging clade, suggesting that this region may be an ancestral center of diversity for *K. malesianus*. Notably, the distribution pattern of the farmed KMAL‐A/KMAL‐A1 provides key insights into its domestication history. Its occurrence across the Philippines, Malaysia, and Indonesia supports its status as a widespread domesticated haplotype and possibly an ancestral lineage from which other haplotypes may have originated. Based on the *COI‐*5P haplotype network, the clustering of closely related wild Malaysian haplotypes around KMAL‐A suggests that it likely originated from wild Malaysian stocks, which were later domesticated and disseminated through anthropogenic activities. Historical records further support this interpretation, noting that seaweed farming practices in the Philippines, particularly in Tawi‐Tawi, may have been influenced by earlier cultivation efforts in Malaysia. Although seaweed farming in Tawi‐Tawi was already established by the mid‐1960s (Hurtado‐Ponce et al. 1996), Eranza et al. (2015) noted that the Bajau and Suluk communities, who have a history of migration between Malaysia and Tawi‐Tawi, likely played a role in transferring farming knowledge and vegetative materials across the Sulu Sea. These early cultivation practices referred generally to *Eucheuma* or *Kappaphycus* (cottonii type) (Hayashi et al. 2017), without species level resolution, which is an important detail because of their high morphological similarity and overlap within the genus. It is therefore possible that the introduced planting materials included the lineage now recognized as KMAL‐A (*COI*‐5P)/MY216 (*cox* 2‐3)/KMAL‐A1 (*COI*‐5P + *cox* 2–3). Interestingly, although KMAL‐A is a farmed haplotype, many of the genetically similar Malaysian haplotypes were collected from the wild. This likely reflects either one of the following: naturalization of feral clones, reproduction and recruitment from farmed cultivar, or a shared ancestral stock thriving in the wild. It is possible that early cultivation of KMAL‐A led to the unintentional dispersal of thallus fragments into the wild, where they established naturalized populations and accumulated minor genetic variations. Alternatively, KMAL‐A and Malaysian wild haplotypes may have both descended independently from a common ancestral population that are naturally distributed across the Philippines, Malaysia, and Indonesia. However, the limited genetic differentiation between them suggests that escape from cultivation is the more plausible explanation.

### 4.3 Conspecificity and Species Complex in *Kappaphycus malesianus* and Allied Taxa

The GUI1 haplotype, classified as an unidentified *Kappaphycus* species, clustered closely with *K. malesianus* haplotypes in multiple gene trees. This suggests a strong likelihood that GUI1 represents a variant or previously unrecognized lineage of *K. malesianus*. This is supported by its consistent grouping with *K. malesianus* haplotypes and high sequence similarity, suggesting conspecificity. Furthermore, KINE‐A and V15, both identified as *K. inermis* , exhibited high homology with *K. malesianus* haplotypes across multiple phylogenetic trees. They clustered closely with *K. malesianus* haplotypes in the phylogenetic analysis despite their divergent positions in the haplotype network. This phylogenetic proximity suggests that *K. inermis* and *K. malesianus* may represent part of a species complex or reflect a historical gene flow and incomplete lineage sorting between these taxa. The deep separation observed in the network further supports the notion of long‐term evolutionary divergence, warranting further investigation into their taxonomic boundaries. The study of Dumilag and Lluisma (2014) also indicates that this remains an unresolved issue and recommends the use of more robust data such as genomic sequences (Crisostomo et al. 2023) to clarify the phylogenetic relationship between these two species.

### 4.4 Conclusion, Limitation, and Recommendation for Genetic Conservation and Future Research

This study reveals that *K. malesianus* has a wider geographic range, greater morphological variability, and higher genetic diversity than previously reported, with new records from Sorsogon, Northern Samar, Bohol, and Iloilo, highlighting distinct regional populations. Phylogeographic analyses indicate a complex population structure shaped by historical gene flow and regional diversification influenced by oceanographic features and plausible anthropogenic activities, which, together, influenced patterns of connectivity, dispersal, and local adaptation across the Philippine archipelago. While the findings provide valuable insights into the genetic diversity and distribution of *K. malesianus* in the Philippines, its generalizability is limited to the geographic scope and number of samples examined in this study. The absence of seascape or environmental analyses prevents a direct assessment of how oceanographic or ecological factors influence population structure. Nonetheless, the results established an important baseline for understanding population connectivity and serve as a foundation for future, broader‐scale assessments. Generally, this study was based solely on the genetic data obtained from a one‐time sampling of *K*. *malesianus* populations, without concurrent environmental measurements or temporal replication. As such, we were unable to perform probabilistic uncertainty assessments (e.g., Global Sensitivity and Uncertainty Analysis or GSUA) or apply non‐linear causal network approaches (e.g., Convergent Cross Mapping) to evaluate eco‐stochasticity or link oceanographic and environmental patterns with ecological processes. Future research that integrates genetic data with environmental parameters and temporal monitoring will be crucial for enabling these systemic analyses, thereby providing deeper insights into ecological drivers and dynamics shaping *K. malesianus* diversity.

Beyond patterns of genetic connectivity, the findings from this study have broader implications for the improvement and conservation of *K. malesianus*. The identification of novel haplotypes not only emphasizes the importance of continued genetic surveys to better understand the identity and evolution of *K. malesianus* but also to help identify strains with traits valuable for seaweed farming. Knowing the wild origins of these strains can guide future breeding programs by tracing genetic diversity in natural populations, which may serve as potential sources of beneficial traits for crop improvement. *K. malesianus* has been reported to exhibit greater resistance to epiphyte infestation compared to its congeners (Ali et al. 2020), making it a promising genetic source for developing epiphyte resistant cultivars. Techniques such as collecting wild carposporophytes (cystocarpic female gametophytes), inducing spore release, and cultivating the resulting germlings (Hinaloc 2017; Hinaloc and Roleda 2021) can also be employed to recover genetically distinct individuals. These can then be screened for desirable biochemical traits (Narvarte et al. 2022) and further evaluated through field trials (Gacura et al. 2026). Furthermore, this study offers new insights into the domestication history and geographic origin of cultivated stocks. This highlights the need to safeguard wild genetic resources by monitoring the movement of farmed seaweeds across regions. Implementing traceability measures, enforcing biosecurity protocols, and promoting the use of diverse locally adapted strains may help reduce the environmental impact of seaweed farming. Overall, the findings of this study emphasize the importance of conserving wild genetic diversity, reducing the risk of genetic uniformity from extensively farmed strains, and expanding genetic surveys to improve our understanding of *K. malesianus* distribution, connectivity, and effective diversity management. Beyond conservation and management (e.g., Gonzaga et al. 2025) of different eucheumatoid taxa, these genetic insights also have practical implications for the carrageenan industry (Mendes et al. 2024; Humayun et al. 2025). The presence of genetically and functionally distinct *K. malesianus* strains in cultivation, particularly when misidentified or mixed with *K. alvarezii*, may contribute to batch‐to‐batch variability in carrageenan quality, an issue already reported by processors sourcing “cottonii” from southern Philippines. Ensuring species‐level identification and traceability is therefore critical for maintaining product consistency and supporting quality assurance.

## Author Contributions

**Ronel T. Aguilar:** conceptualization (equal), data curation (lead), formal analysis (lead), investigation (equal), methodology (equal), visualization (equal), writing – original draft (lead). **Bea A. Crisostomo:** conceptualization (equal), data curation (equal), formal analysis (equal), methodology (equal), validation (equal), writing – review and editing (equal). **Jonh Rey L. Gacura:** conceptualization (equal), investigation (equal), methodology (equal), visualization (equal), writing – review and editing (equal). **Lourie Ann R. Hinaloc:** conceptualization (equal), investigation (equal), methodology (equal), visualization (equal), writing – review and editing (equal). **Michael Y. Roleda:** conceptualization (equal), funding acquisition (lead), methodology (equal), project administration (lead), resources (lead), validation (equal), writing – review and editing (equal).

## Funding

This work was supported by the Commission on Higher Education, The Councils of the Linnean Society of London and the Systematics Association and Philippine Council for Agriculture, Aquatic and Natural Resources Research and Development.

## Conflicts of Interest

The authors declare no conflicts of interest.

## Data Availability

All relevant data, including specimen information and associated metadata, are provided in Table A1. The corresponding nucleotide sequences generated from this study have been deposited in the National Center for Biotechnology Information (NCBI) GenBank, and their accession numbers are summarized in the same table. The sequences can also be accessed directly through NCBI using the following accession ranges: PV876769–PV876809 (*COI*‐5P) https://www.ncbi.nlm.nih.gov/nuccore/?term=PV876769%3APV876809%5Baccn%5D. PV876810–PV876843 (*cox*2‐3) https://www.ncbi.nlm.nih.gov/nuccore/?term=PV876810%3APV876843%5Baccn%5D. PV876844—PV876884 (*rbc*L) https://www.ncbi.nlm.nih.gov/nuccore/?term=PV876844%3APV876884%5Baccn%5D. PV875292—PV875327 (SSU) https://www.ncbi.nlm.nih.gov/nuccore/?term=PV875292%3APV875327%5Baccn%5D.

## Appendix A

**TABLE A1:** List of *Kappaphycus malesianus* collections from the Philippines and supplementary GenBank sequences used in this study.

No.	Isolate	Origin	Collection date	Haplotype					Accession number				References
				*COI*‐5P	*cox*2‐3	*COI*‐5P + *cox*2‐3	*rbc*L	SSU	*COI*‐5P	*cox*2‐3	*rbc*L	SSU	
1	*K. malesianus* 162	Brgy. Dapdap, Bulusan, Sorsogon, Philippines	07‐Oct‐22	KMAL‐H	KMAL‐2	KMAL‐H2	KMAL‐RB2	KMAL‐SS1	PV876804	PV876838	PV876879	PV875323	This study
2	*K. malesianus* 177	Brgy. San Rafael, Sta. Magdalena, Sorsogon, Philippines	08‐Oct‐22	KMAL‐G	KMAL‐3	KMAL‐G3	KMAL‐RB2	—	PV876803	PV876837	PV876878	—	This study
3	*K. malesianus* 178	Brgy. Dancalan, Bulusan, Sorsogon, Philippines	08‐Oct‐22	KMAL‐G	—	—	—	—	PV876802	—	—	—	This study
4	*K. malesianus* 179	Brgy. Dancalan, Bulusan, Sorsogon, Philippines	08‐Oct‐22	KMAL‐G	KMAL‐3	KMAL‐G3	KMAL‐RB2	—	PV876801	PV876836	PV876877	—	This study
5	*K. malesianus* 793	Brgy. Dancalan, Bulusan, Sorsogon, Philippines	08‐Apr‐24	KMAL‐G	KMAL‐2	KMAL‐G2	KMAL‐RB2	KMAL‐SS2	PV876783	PV876824	PV876858	PV875306	This study
6	*K. malesianus* 794	Brgy. Dancalan, Bulusan, Sorsogon, Philippines	08‐Apr‐24	KMAL‐G	KMAL‐2	KMAL‐G2	KMAL‐RB2	KMAL‐SS1	PV876782	PV876823	PV876857	PV875305	This study
7	*K. malesianus 795*	Brgy. Dancalan, Bulusan, Sorsogon, Philippines	08‐Apr‐24	KMAL‐G	KMAL‐2	KMAL‐G2	KMAL‐RB2	KMAL‐SS2	PV876781	PV876822	PV876856	PV875304	This study
8	*K. malesianus 796*	Brgy. Dancalan, Bulusan, Sorsogon, Philippines	08‐Apr‐24	KMAL‐G	KMAL‐2	KMAL‐G2	KMAL‐RB2	KMAL‐SS2	PV876780	PV876821	PV876855	PV875303	This study
9	*K. malesianus* 797	Brgy. Dancalan, Bulusan, Sorsogon, Philippines	08‐Apr‐24	KMAL‐G	KMAL‐2	KMAL‐G2	KMAL‐RB2	KMAL‐SS2	PV876779	PV876820	PV876854	PV875302	This study
10	*K. malesianus* 798	Brgy. Dancalan, Bulusan, Sorsogon, Philippines	08‐Apr‐24	KMAL‐G	KMAL‐2	KMAL‐G2	KMAL‐RB2	KMAL‐SS1	PV876778	PV876819	PV876853	PV875301	This study
11	*K. malesianus 799*	Brgy. Dancalan, Bulusan, Sorsogon, Philippines	08‐Apr‐24	KMAL‐G	KMAL‐2	KMAL‐G2	KMAL‐RB2	KMAL‐SS1	PV876777	PV876818	PV876852	PV875300	This study
12	*K. malesianus* 800	Brgy. Dancalan, Bulusan, Sorsogon, Philippines	08‐Apr‐24	KMAL‐G	KMAL‐2	KMAL‐G2	KMAL‐RB2	KMAL‐SS2	PV876776	PV876817	PV876851	PV875299	This study
13	*K. malesianus* 801	Brgy. Dancalan, Bulusan, Sorsogon, Philippines	08‐Apr‐24	KMAL‐G	KMAL‐2	KMAL‐G2	KMAL‐RB2	KMAL‐SS1	PV876775	PV876816	PV876850	PV875298	This study
14	*K. malesianus 802*	Brgy. Dancalan, Bulusan, Sorsogon, Philippines	08‐Apr‐24	KMAL‐G	KMAL‐2	KMAL‐G2	KMAL‐RB2	KMAL‐SS2	PV876774	PV876815	PV876849	PV875297	This study
15	*K. malesianus* 803	Brgy. Dancalan, Bulusan, Sorsogon, Philippines	08‐Apr‐24	KMAL‐G	KMAL‐2	KMAL‐G2	KMAL‐RB2	KMAL‐SS2	PV876773	PV876814	PV876848	PV875296	This study
16	*K. malesianus* 804	Brgy. Dancalan, Bulusan, Sorsogon, Philippines	08‐Apr‐24	KMAL‐G	KMAL‐2	KMAL‐G2	KMAL‐RB2	KMAL‐SS2	PV876772	PV876813	PV876847	PV875295	This study
17	*K. malesianus* 805	Brgy. Dancalan, Bulusan, Sorsogon, Philippines	08‐Apr‐24	KMAL‐G	KMAL‐2	KMAL‐G2	KMAL‐RB2	KMAL‐SS2	PV876771	PV876812	PV876846	PV875294	This study
18	*K. malesianus* 806	Brgy. Dancalan, Bulusan, Sorsogon, Philippines	08‐Apr‐24	KMAL‐G	KMAL‐2	KMAL‐G2	KMAL‐RB2	KMAL‐SS2	PV876770	PV876811	PV876845	PV875293	This study
19	*K. malesianus* 807	Brgy. Dancalan, Bulusan, Sorsogon, Philippines	08‐Apr‐24	KMAL‐G	KMAL‐2	KMAL‐G2	KMAL‐RB2	KMAL‐SS2	PV876769	PV876810	PV876844	PV875292	This study
20	*K. malesianus* 150	Brgy. Barobaybay, Lavezares, Northern Samar, Philippines	06‐Sep‐22	KMAL‐G	KMAL‐2	KMAL‐G2	KMAL‐RB2	KMAL‐SS3	PV876809	PV876843	PV876884	PV875327	This study
21	*K. malesianus* 151	Brgy. Barobaybay, Lavezares, Northern Samar, Philippines	06‐Sep‐22	KMAL‐G	KMAL‐2	KMAL‐G2	KMAL‐RB2	—	PV876808	PV876842	PV876883	—	This study
22	*K. malesianus* 152	Brgy. Barobaybay, Lavezares, Northern Samar, Philippines	06‐Sep‐22	KMAL‐G	KMAL‐2	KMAL‐G2	KMAL‐RB2	KMAL‐SS3	PV876807	PV876841	PV876882	PV875326	This study
23	*K. malesianus* 153	Brgy. Barobaybay, Lavezares, Northern Samar, Philippines	06‐Sep‐22	KMAL‐G	KMAL‐2	KMAL‐G2	KMAL‐RB2	KMAL‐SS1	PV876806	PV876840	PV876881	PV875325	This study
24	*K. malesianus* 154	Brgy. Barobaybay, Lavezares, Northern Samar, Philippines	06‐Sep‐22	KMAL‐G	KMAL‐2	KMAL‐G2	KMAL‐RB2	KMAL‐SS1	PV876805	PV876839	PV876880	PV875324	This study
25	*K. malesianus* 553	Brgy. Barobaybay, Lavezares, Northern Samar, Philippines	04‐Sep‐23	KMAL‐G	—	—	KMAL‐RB2	KMAL‐SS1	PV876795	—	PV876871	PV875318	This study
26	*K. malesianus* 555	Brgy. Barobaybay, Lavezares, Northern Samar, Philippines	04‐Sep‐23	KMAL‐G	—	—	KMAL‐RB2	—	PV876794	—	PV876870	—	This study
27	*K. malesianus* 556	Brgy. Barobaybay, Lavezares, Northern Samar, Philippines	04‐Sep‐23	KMAL‐G	—	—	KMAL‐RB2	KMAL‐SS1	PV876793	—	PV876869	PV875317	This study
28	*K. malesianus* 557	Brgy. Barobaybay, Lavezares, Northern Samar, Philippines	04‐Sep‐23	KMAL‐G	KMAL‐4	KMAL‐G4	KMAL‐RB2	KMAL‐SS1	PV876792	PV876830	PV876868	PV875316	This study
29	*K. malesianus* 558	Brgy. Barobaybay, Lavezares, Northern Samar, Philippines	04‐Sep‐23	KMAL‐G	KMAL‐2	KMAL‐G2	KMAL‐RB2	KMAL‐SS1	PV876791	PV876829	PV876867	PV875315	This study
30	*K. malesianus* 559	Brgy. Barobaybay, Lavezares, Northern Samar, Philippines	04‐Sep‐23	KMAL‐G	—	—	KMAL‐RB2	KMAL‐SS1	PV876790	—	PV876866	PV875314	This study
31	*K. malesianus* 560	Brgy. Barobaybay, Lavezares, Northern Samar, Philippines	04‐Sep‐23	KMAL‐G	KMAL‐2	KMAL‐G2	KMAL‐RB2	KMAL‐SS1	PV876789	PV876828	PV876865	PV875313	This study
32	*K. malesianus* 561	Brgy. Barobaybay, Lavezares, Northern Samar, Philippines	04‐Sep‐23	KMAL‐G	—	—	KMAL‐RB2	KMAL‐SS1	PV876788	—	PV876864	PV875312	This study
33	*K. malesianus* 562	Brgy. Barobaybay, Lavezares, Northern Samar, Philippines	04‐Sep‐23	KMAL‐G	KMAL‐2	KMAL‐G2	KMAL‐RB2	KMAL‐SS1	PV876787	PV876827	PV876863	PV875311	This study
34	*K. malesianus* 565	Brgy. Barobaybay, Lavezares, Northern Samar, Philippines	04‐Sep‐23	KMAL‐G	—	—	KMAL‐RB2	KMAL‐SS1	PV876786	—	PV876862	PV875310	This study
35	*K. malesianus* 567	Brgy. Barobaybay, Lavezares, Northern Samar, Philippines	04‐Sep‐23	KMAL‐G	KMAL‐2	KMAL‐G2	KMAL‐RB2	KMAL‐SS1	PV876785	PV876826	PV876861	PV875309	This study
36	*K. malesianus* 280	Danajon Bank, Bohol, Philippines	06‐Jan‐23	KMAL‐I	GUI1	KMAL‐I1	KMAL‐RB3	KMAL‐SS1	PV876800	PV876835	PV876876	PV875322	This study
37	*K. malesianus* 313	Danajon Bank, Bohol, Philippines	06‐Jan‐23	KMAL‐I	GUI1	KMAL‐I1	KMAL‐RB3	KMAL‐SS1	PV876799	PV876834	PV876875	PV875321	This study
38	*K. malesianus* 326	Danajon Bank, Bohol, Philippines	06‐Jan‐23	KMAL‐I	GUI1	KMAL‐I1	KMAL‐RB3	—	PV876798	PV876833	PV876874	—	This study
39	*K. malesianus* 375	Danajon Bank, Bohol, Philippines	07‐Jan‐23	KMAL‐J	GUI1	KMAL‐J1	KMAL‐RB3	KMAL‐SS1	PV876797	PV876832	PV876873	PV875320	This study
40	*K. malesianus* 640	Danajon Bank, Bohol, Philippines	01‐Oct‐23	KMAL‐I	GUI1	KMAL‐I1	KMAL‐RB3	KMAL‐SS1	PV876784	PV876825	PV876860	PV875308	This study
41	*K. malesianus* 642	Danajon Bank, Bohol, Philippines	01‐Oct‐23	—	—	—	KMAL‐RB3	KMAL‐SS1	—	—	PV876859	PV875307	This study
42	*K. malesianus* 407	Brgy. Pili, Ajuy, Iloilo, Philippines	11‐Mar‐23	GUI1	ILO1	ILO1	KMAL‐RB3	KMAL‐SS1	PV876796	PV876831	PV876872	PV875319	This study

			Haplotype					Accession number				References
			*COI*‐5P	*cox*2‐3	*COI*‐5P + *cox*2‐3	*rbc*L	SSU	*COI*‐5P	*cox*2‐3	*rbc*L	SSU	
*Additional GenBank sequences used for haplotype and phylogenetic analyses of mitochondrial markers (COI‐5P and cox2‐3)*												
43	*K. malesianus* “Aring‐Aring” 49	Malaysia, Philippines, and Indonesia	KMAL‐A	MY216	KMAL‐A1			JX624032	JN663785			Tan et al. (2012); Lim et al. (2014)
44	*K. malesianus* “Kalog Kalog/Bulate” TAW 107	Tinambak, Tongmageng, Sitangkai, Tawi‐Tawi, Philippines	KMAL‐B	KMAL‐1	KMAL‐B1			KT316584	KT316637			Dumilag et al. (2016)
45	*K. malesianus* “Kalog Kalog/Bulate” TAW 107	Tinambak, Tongmageng, Sitangkai, Tawi‐Tawi, Philippines	KMAL‐B	MY216	KMAL‐B2			KT316582	KT316635			Dumilag et al. (2016)
46	*K. malesianus* RD122	Tihikan, Tondon, Panglima Sugala, Tawi‐Tawi, Philippines	KMAL‐C	—	—			ON858744	—			Dumilag et al. (2023)
47	*K. malesianus* RD150	Tihikan, Tondon, Panglima Sugala, Tawi‐Tawi, Philippines	KMAL‐D	—	—			ON858770	—			Dumilag et al. (2023)
48	*K. malesianus* RD165	Tihikan, Tondon, Panglima Sugala, Tawi‐Tawi, Philippines	KMAL‐E	—	—			ON858779	—			Dumilag et al. (2023)
49	*K. malesianus* RD131	Tihikan, Tondon, Panglima Sugala, Tawi‐Tawi, Philippines	KMAL‐F	—	—			ON858752	—			Dumilag et al. (2023)
50	*K. malesianus* 92	Sabangkat, Sabah, Malaysia	MY92	—	—			KC905317	—			Tan et al. (2014)
51	*K. malesianus* 93	Sabangkat, Sabah, Malaysia	MY93	—	—			JX624033	—			Tan et al. (2012)
52	*K. malesianus* OMD1	Omadal, Sabah, Malaysia	OMD1	—	—			OL841264	—			Tan et al. (2022)
53	*K. malesianus* OND43	Omadal, Sabah, Malaysia	OMD43	—	—			OL841295	—			Tan et al. (2022)
54	*K. malesianus* SGM5	Singgah Mata, Sabah, Malaysia	SGM5	—	—			OL841196	—			Tan et al. (2022)
55	*Kappaphycus* sp. 1 PEL‐2013	Guimaras Island, Panay, Philippines	GUI1	GUI1	GUI1			KC905320	KC905430			Lim et al. (2014)
56	*K. inermis*	Truong Sa Island, Khanh Hoa, Vietnam	V15	V15	V15			KC905321	KC905431			Lim et al. (2014); Dumilag et al. (2023)
57	*K. inermis* RVD10016	Burgos, Ilocos Norte, Philippines	KINE‐A	KINE‐1	KINE‐A1			KT316578	KT316629			Dumilag and Lluisma (2014)
58	*K.alvarezii* SUR 101	Balibadon, Cortes, Surigao del Sur, PH	KALV‐A	KALV‐1	KALV‐A1			KT316568	KT316616			Dumilag et al. (2016)
59	*K. striatus* ZAM 106	Sacol Strait, Zamboanga sel Sur, PH	KSTR‐A	89	KSA			KT316602	KT316659			Dumilag et al. (2016)
60	*K. cottonii* KCOT2‐10	Dancalan, Bulusan, Sorsogon	KCOT‐A	KCOT‐1	KCOT‐A1			MF134475	MF134574			Dumilag et al. (2018)
61	*E. denticulatum* BOH2	Bohol, Central Visayas, Philippines	EDA	13	EDA‐13			KC905336	KC905445			Lim et al. (2014)
*Additional GenBank sequences used for haplotype and phylogenetic analyses of rbcL sequences*												
62	*K. malesianus* 218	Karindingan, Sabah, Malaysia				KMAL‐RB1				KC905220		Tan et al. (2014)
63	*K. malesianus* 92	Sabangkat, Sabah, Malaysia				KMAL‐RB1				KC905226		Tan et al. (2014)
64	*K. malesianus* P1	Pulau Panjang, Papua, Malaysia				KMAL‐RB1				KC905228		Tan et al. (2014)
65	*Kappaphycus* sp. “Aring‐Aring” 49	Malaysia				KMAL‐RB1				JX624003		Tan et al. (2012)
66	*Kappaphycus* sp. “Aring‐Aring” 93	Malaysia				KMAL‐RB1				JX624004		Tan et al. (2012)
67	*Kappaphycus* sp. “Aring‐Aring” 115	Malaysia				KMAL‐RB1				JX624005		Tan et al. (2012)
68	*K. alvarezii* 58	Malaysia				KA1				JX623985		Tan et al. (2012)
69	*K. striatus* 1	Malaysia				KS1				JX623992		Tan et al. (2012)
70	*E. denticulatum* BOH5	Philippines				ED1				JX624010		Tan et al. (2012)
*Additional GenBank sequences used for haplotype and phylogenetic analyses of SSU sequences*												
71	*K. alvarezii*	Philippines					KALV (a)^a^				U25437	Lluisma and Ragan (1995)
72	*K. alvarezii* TN	Malaysia					KALV (b)^a^				JN984917	Thien et al. (2011c)
73	*K. alvarezii* GT	Malaysia					KALV (c)^a^				JN984918	Thien et al. (2011f)
74	*K. alvarezii* BA	Malaysia					KALV (d)^a^				JQ388229	Thien et al. (2011a )
75	*K. alvarezii* BN	Malaysia					KALV (e)^a^				JQ388230	Thien et al. (2011b)
76	*K. alvarezii* EK01	Rameshwaram, Tamil Nadu, India					KALV (f)^a^				KT799800	Biji and Eswaran (2015)
77	*K. striatus* AG	Malaysia					KSTR (a)^a^				JN984919	Thien et al. (2011d)
78	*K. striatus* GF	Malaysia					KSTR (b)^a^				JN984920	Thien et al. (2011e)
79	*K. striatus* YF	Malaysia					KSTR (c)^a^				JN984921	Thien et al. (2011g)
80	*E. denticulatum*	Philippines					—				U25439	Lluisma and Ragan (1995)
*Additional Kapappahycus malesianus sequences from the Philippines used for DnaSP analysis*												
81	“Halaw” TAW 100	Tinambak, Tongmageng, Sitangkai, Tawi‐Tawi, Philippines	—	MY216	—			—	KT316633			Dumilag et al. (2016)
82	“Kuku Belleh” TAW 101	Tinambak, Tongmageng, Sitangkai, Tawi‐Tawi, Philippines	—	MY216	—			—	KT316634			Dumilag et al. (2016)
83	“Kalog Kalog/Bulate” TAW 107	Tinambak, Tongmageng, Sitangkai, Tawi‐Tawi, Philippines	KMAL‐B	MY216	KMAL‐B2			KT316582	KT316635			Dumilag et al. (2016)
84	“Kalog Kalog/Bulate” TAW 108	Tinambak, Tongmageng, Sitangkai, Tawi‐Tawi, Philippines	KMAL‐B	MY216	KMAL‐B2			KT316583	KT316636			Dumilag et al. (2016)
85	“Kalog Kalog/Bulate” TAW 109	Tinambak, Tongmageng, Sitangkai, Tawi‐Tawi, Philippines	KMAL‐B	KMAL‐1	KMAL‐B1			KT316584	KT316637			Dumilag et al. (2016)
86	“Kalog Kalog/Bulate” TAW 110	Tinambak, Tongmageng, Sitangkai, Tawi‐Tawi, Philippines	KMAL‐B	MY216	KMAL‐B2			KT316585	KT316638			Dumilag et al. (2016)
87	“Kalog Kalog/Bulate” TAW 111	Tinambak, Tongmageng, Sitangkai, Tawi‐Tawi, Philippines	—	MY216	—			—	KT316639			Dumilag et al. (2016)
88	“Kalog Kalog/Bulate” TAW 112	Tinambak, Tongmageng, Sitangkai, Tawi‐Tawi, Philippines	KMAL‐B	MY216	KMAL‐B2			KT316586	KT316640			Dumilag et al. (2016)
89	“Kalog Kalog/Bulate” TAW 113	Tinambak, Tongmageng, Sitangkai, Tawi‐Tawi, Philippines	KMAL‐B	MY216	KMAL‐B2			KT316587	KT316641			Dumilag et al. (2016)
90	“Kalog Kalog/Bulate” TAW 114	Tinambak, Tongmageng, Sitangkai, Tawi‐Tawi, Philippines	KMAL‐B	MY216	KMAL‐B2			KT316588	KT316642			Dumilag et al. (2016)
91	“Kalog Kalog/Bulate” TAW 115	Tinambak, Tongmageng, Sitangkai, Tawi‐Tawi, Philippines	KMAL‐B	MY216	KMAL‐B2			KT316589	KT316643			Dumilag et al. (2016)
92	“Kalog Kalog/Bulate” TAW 116	Tinambak, Tongmageng, Sitangkai, Tawi‐Tawi, Philippines	KMAL‐B	—	—			KT316590	—			Dumilag et al. (2016)
93	“Kalog Kalog/Bulate” TAW 117	Tinambak, Tongmageng, Sitangkai, Tawi‐Tawi, Philippines	—	MY216	—			—	KT316644			Dumilag et al. (2016)
94	*K. malesianus* RD121	Tihikan, Tondon, Panglima, Sugala, Tawi‐Tawi, Philippines	KMAL‐C	—	—			ON858743	—			Dumilag et al. (2023)
95	*K. malesianus* RD122	Tihikan, Tondon, Panglima, Sugala, Tawi‐Tawi, Philippines	KMAL‐C	—	—			ON858744	—			Dumilag et al. (2023)
96	*K. malesianus* RD123	Tihikan, Tondon, Panglima, Sugala, Tawi‐Tawi, Philippines	KMAL‐C	—	—			ON858745	—			Dumilag et al. (2023)
97	*K. malesianus* RD124	Tihikan, Tondon, Panglima, Sugala, Tawi‐Tawi, Philippines	KMAL‐C	—	—			ON858746	—			Dumilag et al. (2023)
98	*K. malesianus* RD128	Tihikan, Tondon, Panglima, Sugala, Tawi‐Tawi, Philippines	KMAL‐C	—	—			ON858749	—			Dumilag et al. (2023)
99	*K. malesianus* RD129	Tihikan, Tondon, Panglima, Sugala, Tawi‐Tawi, Philippines	KMAL‐C	—	—			ON858750	—			Dumilag et al. (2023)
100	*K. malesianus* RD130	Tihikan, Tondon, Panglima, Sugala, Tawi‐Tawi, Philippines	KMAL‐C	—	—			ON858751	—			Dumilag et al. (2023)
101	*K. malesianus* RD131	Tihikan, Tondon, Panglima, Sugala, Tawi‐Tawi, Philippines	KMAL‐F	—	—			ON858752	—			Dumilag et al. (2023)
102	*K. malesianus* RD132	Tihikan, Tondon, Panglima, Sugala, Tawi‐Tawi, Philippines	KMAL‐C	—	—			ON858753	—			Dumilag et al. (2023)
103	*K. malesianus* RD134	Tihikan, Tondon, Panglima, Sugala, Tawi‐Tawi, Philippines	KMAL‐C	—	—			ON858755	—			Dumilag et al. (2023)
104	*K. malesianus* RD135	Tihikan, Tondon, Panglima, Sugala, Tawi‐Tawi, Philippines	KMAL‐C	—	—			ON858756	—			Dumilag et al. (2023)
105	*K. malesianus* RD136	Tihikan, Tondon, Panglima, Sugala, Tawi‐Tawi, Philippines	KMAL‐C	—	—			ON858757	—			Dumilag et al. (2023)
106	*K. malesianus* RD137	Tihikan, Tondon, Panglima, Sugala, Tawi‐Tawi, Philippines	KMAL‐C	—	—			ON858758	—			Dumilag et al. (2023)
107	*K. malesianus* RD138	Tihikan, Tondon, Panglima, Sugala, Tawi‐Tawi, Philippines	KMAL‐C	—	—			ON858759	—			Dumilag et al. (2023)
108	*K. malesianus* RD139	Tihikan, Tondon, Panglima, Sugala, Tawi‐Tawi, Philippines	KMAL‐C	—	—			ON858760	—			Dumilag et al. (2023)
109	*K. malesianus* RD141	Tihikan, Tondon, Panglima, Sugala, Tawi‐Tawi, Philippines	KMAL‐C	—	—			ON858762	—			Dumilag et al. (2023)
110	*K. malesianus* RD143	Tihikan, Tondon, Panglima, Sugala, Tawi‐Tawi, Philippines	KMAL‐C	—	—			ON858763	—			Dumilag et al. (2023)
111	*K. malesianus* RD145	Tihikan, Tondon, Panglima, Sugala, Tawi‐Tawi, Philippines	KMAL‐C	—	—			ON858765	—			Dumilag et al. (2023)
112	*K. malesianus* RD146	Tihikan, Tondon, Panglima, Sugala, Tawi‐Tawi, Philippines	KMAL‐C	—	—			ON858766	—			Dumilag et al. (2023)
113	*K. malesianus* RD147	Tihikan, Tondon, Panglima, Sugala, Tawi‐Tawi, Philippines	KMAL‐C	—	—			ON858767	—			Dumilag et al. (2023)
114	*K. malesianus* RD148	Tihikan, Tondon, Panglima, Sugala, Tawi‐Tawi, Philippines	KMAL‐C	—	—			ON858768	—			Dumilag et al. (2023)
115	*K. malesianus* RD149	Tihikan, Tondon, Panglima, Sugala, Tawi‐Tawi, Philippines	KMAL‐C	—	—			ON858769	—			Dumilag et al. (2023)
116	*K. malesianus* RD150	Tihikan, Tondon, Panglima, Sugala, Tawi‐Tawi, Philippines	KMAL‐C	—	—			ON858770	—			Dumilag et al. (2023)
117	*K. malesianus* RD153	Tihikan, Tondon, Panglima, Sugala, Tawi‐Tawi, Philippines	KMAL‐C	—	—			ON858772	—			Dumilag et al. (2023)
118	*K. malesianus* RD156	Tihikan, Tondon, Panglima, Sugala, Tawi‐Tawi, Philippines	KMAL‐C	—	—			ON858774	—			Dumilag et al. (2023)
119	*K. malesianus* RD157	Tihikan, Tondon, Panglima, Sugala, Tawi‐Tawi, Philippines	KMAL‐C	—	—			ON858775	—			Dumilag et al. (2023)
120	*K. malesianus* RD162	Tihikan, Tondon, Panglima, Sugala, Tawi‐Tawi, Philippines	KMAL‐D	—	—			ON858777	—			Dumilag et al. (2023)
121	*K. malesianus* RD164	Tihikan, Tondon, Panglima, Sugala, Tawi‐Tawi, Philippines	KMAL‐C	—	—			ON858778	—			Dumilag et al. (2023)
122	*K. malesianus* RD165	Tihikan, Tondon, Panglima, Sugala, Tawi‐Tawi, Philippines	KMAL‐E	—	—			ON858779	—			Dumilag et al. (2023)
123	*K. malesianus* RD168	Tihikan, Tondon, Panglima, Sugala, Tawi‐Tawi, Philippines	KMAL‐C	—	—			ON858781	—			Dumilag et al. (2023)
124	*K. malesianus* RD1528	Landang, Laum, Sacol Island, Zamboanga City, Philippines	KMAL‐A	—	—			ON858784	—			Dumilag et al. (2023)

^a^ Assigned letters in parentheses are representative labels and should not be interpreted as haplotype designations.
